# Structural relaxation phenomena in silicate glasses modified by irradiation with femtosecond laser pulses

**DOI:** 10.1038/srep43815

**Published:** 2017-03-07

**Authors:** Thomas Seuthe, Alexandre Mermillod-Blondin, Moritz Grehn, Jörn Bonse, Lothar Wondraczek, Markus Eberstein

**Affiliations:** 1 Fraunhofer IKTS, Fraunhofer Institute for Ceramic Technologies and Systems, Winterbergstraße 28, 01277 Dresden, Germany; 2 Max-Born-Institute for Nonlinear Optics and Short Pulse Spectroscopy, Max-Born-Straße 2a, 12489 Berlin, Germany; 3 Technische Universität Berlin, Department of Optics and Atomic Physics, Straße des 17. Juni 135, 10623 Berlin, Germany; 4 Bundesanstalt für Materialforschung und –prüfung (BAM), Unter den Eichen 87, 12205 Berlin, Germany; 5 Otto-Schott-Institute of Materials Research, Fraunhoferstraße 6, 07743 Jena, Germany

## Abstract

Structural relaxation phenomena in binary and multicomponent lithium silicate glasses were studied upon irradiation with femtosecond (fs) laser pulses (800 nm central wavelength, 130 fs pulse duration) and subsequent thermal annealing experiments. Depending on the annealing temperature, micro-Raman spectroscopy analyses evidenced different relaxation behaviours, associated to bridging and non-bridging oxygen structures present in the glass network. The results indicate that the mobility of lithium ions is an important factor during the glass modification with fs-laser pulses. Quantitative phase contrast imaging (spatial light interference microscopy) revealed that these fs-laser induced structural modifications are closely related to local changes in the refractive index of the material. The results establish a promising strategy for tailoring fs-laser sensitivity of glasses through structural mobility.

The physical mechanisms governing the laser matter interaction with dielectrics are manifold^1^. Their sequence begins with nonlinear absorption of the laser radiation, resulting in the ultrafast formation of an electron-hole plasma confined in the solid^2^,^3^. Electron-phonon coupling transfers the electronic energy to the lattice, provoking a temperature increase and local melting of the glass^4^. In the melt, structural disorder appears as the ions gain mobility. The presence of a hot volume reaching temperatures up to several thousands of Kelvin^5^ surrounded by a cold environment results in huge spatial temperature gradients. As a consequence, cooling at rates as high as 10^8^ Ks^−1^, the glass constituents ‘freeze’ in their actual configuration, determining the structural state of the fs-laser modified material. Note that these cooling rates exceed by several orders of magnitude what is usually obtained by conventional manufacturing techniques^5^. The fs-laser induced structural modification translates into a refractive index change which is the cornerstone of a multitude of applications such as optical waveguides^6^,^7^, optical couplers^8^,^9^, or multiplexers for micro-holographic data storage^10^.

Several approaches have been employed to study fs-laser induced structural and/or chemical modifications. X-ray absorption near edge structure (XANES) spectroscopy has proven to be successful in estimating specific bond length variations in laser-irradiated potassium-magnesium silicate glasses^11^. Elemental mapping using energy dispersive X-ray analysis (EDX or EPMA) enabled the visualization of spatial ion migration in several glasses^12^,^13^. In the optical domain, Raman spectroscopy is known as a powerful tool to analyse structural changes in amorphous solids (such as glasses) with respect to their composition^14^,^15^,^16^,^17^,^18^,^19^. Moreover, micro-Raman spectroscopy was successfully employed to investigate fs-laser-induced material modifications in different glasses^20^,^21^.

In this work, we take benefit of the structural sensitivity of micro-Raman spectroscopy along with thermal annealing at various temperatures above (see the Supplementary Information) and below the glass transformation temperature (*T*_g_) to probe the structural relaxation phenomena in two distinct fs-laser irradiated lithium silicate glasses, namely LS (24 mol% Li_2_O and 76 mol% SiO_2_) and LNMS (13 mol% Li_2_O, 15 mol% Na_2_O, 9 mol% MgO and 63 mol% SiO_2_). The binary lithium silicate glass (LS) and the multicomponent glass (LNMS) were selected for this study as they both exhibit characteristic changes in the local Si-O-Si bond angles and in the Si-O* bond strengths upon fs-laser induced material modification^22^. Those modifications can be directly probed via micro Raman spectroscopy and allow a qualification of the structural state of the material. Different annealing behaviours (associated with α- and β-relaxations processes) were identified above and below *T*_g_. Complementary quantitative phase contrast imaging measurements reveal the correlation between structural arrangement and refractive index.

## Results

### Raman spectra of the pristine glasses

**Figure 1:**
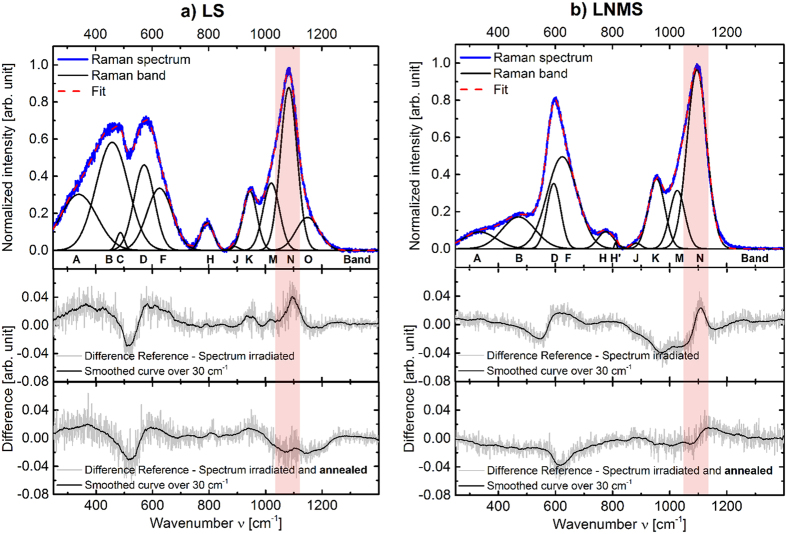
Baseline-corrected and normalized Raman spectra of the glasses (blue solid lines) LS (**a**) and LNMS (**b**) before modification with a fs-laser pulse. These reference spectra are deconvoluted into several individual bands with Gaussian shape (black solid lines) for evaluation of changes and the resultant fit (red dashed line). Middle: Spectral changes after irradiation obtained by subtracting the spectra measured before and after irradiation. Bottom: Spectral changes after irradiation and annealing obtained by subtracting the spectra measured before and after irradiation and thermal treatment. The annealing times and temperatures were 240 min at 0.81 × *T*_g_ (LS) and 60 min at 0.94 × *T*_g_ (LNMS).

Figure 1 (top) shows the baseline-corrected and normalized Raman spectra (blue solid lines) of the non-irradiated glasses LS (a) and LNMS (b) determined experimentally. The black solid lines represent specific Raman bands associated to individual stretching and bending motions of specific sub-structures in silicate glasses. With the exception of two defect types (D1, D2, associated with bands C and E), those sub-structures are called Q^n^-structural elements, where 0 ≤ n ≤ 4 is the number of bridging oxygen ions per tetrahedral cell^23^. The red dashed lines are the least-squares-fitted linear combination of all bands used for deconvolution (see Table 1). These reference spectra can be split into two sections. The low-frequency section from 200 cm^−1^ to about 850 cm^−1^ is attributed mostly to bending vibrations of various glass sub-structures (bands A to H). The most interesting band within this section is band B, centred at about 450 cm^−1^, which is associated to Si-O-Si bending vibrations. Specifically, changes of the centre position of band B reflects changes in the Si-O-Si angle^14^,^24^. Additionally it has to be mentioned, that band C, associated with 4-membered rings of SiO_4_ tetrahedra is, in theory, not expected since there should be sufficient cations to prevent their formation at the LS composition. However, for the deconvolution of the Raman spectra it was necessary to include band C in order to receive appropriate fits. Hence, small amounts of 4-membered rings of SiO_4_ tetrahedra appear to be present in the LS glass, supposedly due to statistical variations in the composition of the amorphous glass network. In the Supplementary Information to this article we provide a study of the relaxation dynamics upon annealing of laser-induced structural modifications above *T*_g_ (α-relaxation^25^) based on the analysis of band B. In what follows, we focus on the study of the high-frequency section between 850 cm^−1^ and 1400 cm^−1^. This frequency range corresponds to stretching vibrations of sub-structures in the glass containing non-bridging oxygen ions (bands J to O). In this part of the spectrum, band N (centred around 1080 cm^−1^) is the most pronounced spectral contribution. The Raman N band was selected for further analysis here, since it exhibits the largest amplitude with only few overlap of other Raman bands. In an earlier work^22^, we have shown that a negative shift of this band indicates a weakening of the Si-O* bond strength.

### Raman spectra of fs-laser irradiated glasses

**Figure 2:**
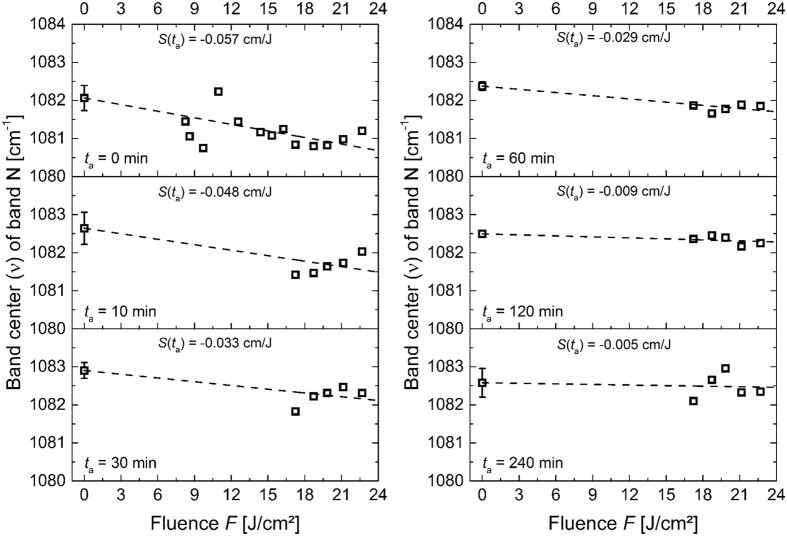
Raman band (N) position *ν* measured in the centre of fs-laser ablation spots on the LS glass sample as function of the peak fluence *F* upon thermal annealing at 0.81 × *T*_g_ (600 K) after *t*_a_ = 0 min (reference, top left), 10 min, 30 min, 60 min, 120 min and 240 min (bottom right). *S(t*_a_) was used to characterize the degree of modification. The data of the curve for *t*_a_ = 0 min are taken from ref. 22.

The LS glass sample was irradiated by single near-infrared 130-fs-laser laser pulses with peak fluences *F* up to 22.5 J/cm^2^ (see Methods section for additional details). Subsequently, the sample was thermally annealed at 0.81 × *T*_g_ (600 K) for various annealing times *t*_a_ (0 ≤ *t*_a_ ≤ 240 minutes). The corresponding spectral changes after irradiation and after annealing are shown in Fig. 1a (middle and bottom, respectively). For the five biggest ablation craters (the 5 largest fluences), Raman spectra were acquired in the centre of the ablation spot, baseline corrected, normalized, and deconvoluted. In Fig. 2, the value of the band N centre position (ν) is plotted versus the fluence (*F*) for various *t*_a_’s. Immediately after irradiation (*t*_a_ = 0), the band N position shifts towards lower wavenumbers from 1082.5 cm^−1^ down to approximately 1081.5 cm^−1^ as *F* increases. Interestingly, for fluences exceeding ~19 J/cm^2^ and *t*_a_ < 30 min, the band N position shifts again toward positive wavenumber values. This can be attributed to a “self-annealing effect”: due to the low thermal conductivity of the glass the amount of deposited energy cannot immediately dissipate via heat into the surrounding. Hence, the cooling of the laser-excited region is slow enough for the glass to relax already partly during the laser-induced cooling stage. Self-annealing is not detectable for *t*_a_ > 30 min. As a consequence, only the slopes *S(t*_a_ = 0 min) and *S(t*_a_ = 10 min) may be underestimated. Furthermore, we emphasize that for *t*_a_ = 0 min, the contribution of self-annealing to the estimate of *S(t*_a_) is mitigated by the availability of numerous data points in the fluence range <19 J/cm^2^, where no self-annealing occurs.

In order to determine the evolution of the band N position (*ν*) as a function of the Fluence (*F*), a linear regression with a slope *S(t*_a_) = d*ν*/d*F* was applied. As a result, we obtain a value of *S(t*_a_) = −0.057 cm/J, indicating a weakening of the Si-O* bond strength.

As the thermal annealing time increases, smaller values of *S(t*_a_) are obtained, down to −0.005 cm/J after a *t*_a_ of 240 minutes. Although the Raman shifts are very small and just above the instrumental precision (see error bars for *F* = 0 J/cm^2^ in Fig. 2), the systematic decrease of *S(t*_a_) is consistent with a progressive relaxation of the fs-laser induced structural modifications upon thermal annealing. This relaxation is further evidenced by plotting the spectral changes after irradiation and annealing, as shown in Fig. 1a. In this figure, the evolution of band N (red emphasized in Fig. 1) after irradiation shows up as a well-contrasted peak (see Fig. 1a, middle) which disappears after 240 min annealing (see Fig. 1a, bottom).

### Structural relaxation below *T*g (β-relaxation)

**Figure 3:**
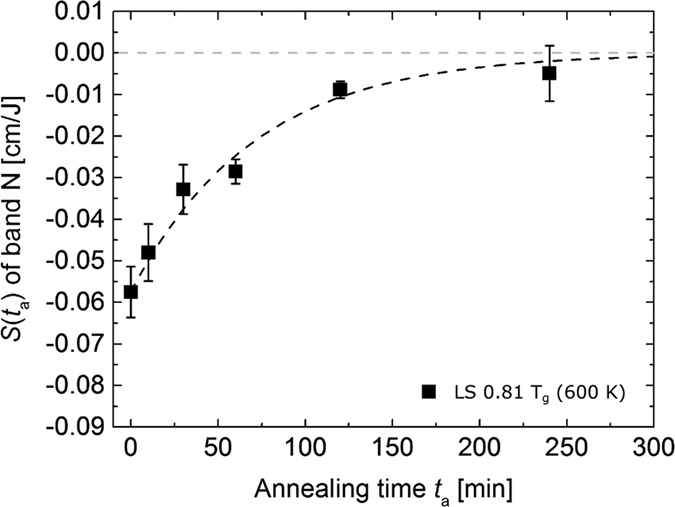
*S(t*_a_) for Raman band N (Si-O* Q^3^ stretching vibrations) measured in the centre of a fs-laser induced ablation spot on the LS glass sample as function of the annealing time *t*_a_, as taken from Fig. 2. The grey dashed horizontal line indicates no modification. The black dashed line guides the eye.

To visualize the changes in the degree of fs-laser induced structural modifications, the slopes *S(t*_a_) determined from Fig. 2 are plotted against the annealing time in Fig. 3. Note that after an annealing time of 240 min at 0.81 × *T*_g_, the degree of modification of the Si-O* structures converges to the initial value of the pristine glass.

For a closer investigation of the relaxation behaviour, an analytical approach used by Wondraczek *et al*.^26^ was applied. For that, the normalized relaxation progress *ξ(t*_a_) was introduced as


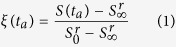


where *S(t*_a_) represents the slope of the linear regression after a particular annealing time *t*_a_, 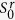 is the slope of the linear regression at a maximum amount of laser-induced modification (here considered as the reference value after an annealing time of *t*_a_ = 0), and 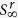 is the slope of the linear regression after an infinite annealing time (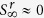).

**Figure 4:**
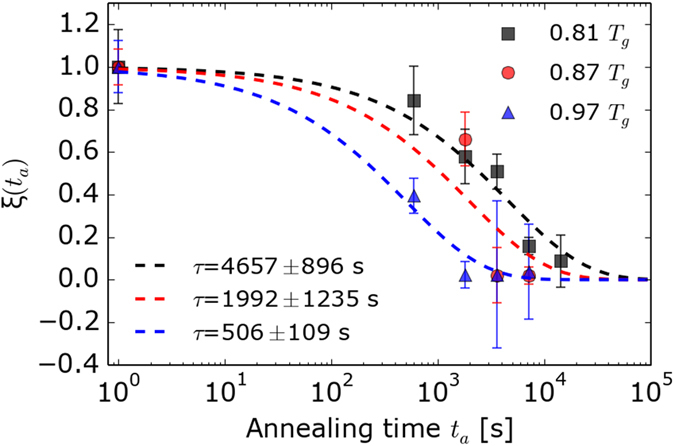
Evolution of the relaxation parameter *ξ* as a function of the annealing time *t*_a_ for 0.81 × *T*_g_ (squares), 0.87 × *T*_g_ (circles) and 0.97 × *T*_g_ (triangles). The dashed lines represent least-squares fits calculated using Eq. (2).

In Fig. 4, the values of the normalized relaxation progress *ξ(t*_a_) are plotted against the annealing time *t*_a_. A least-squares fit with a stretched exponential function (Eq. 2) was used to obtain the characteristic relaxation time *τ*:


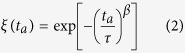


The Kohlrausch exponent *β* was fixed at 0.6, as used by other authors before, to evaluate thermally-induced structural relaxation in glasses^26^,^27^,^28^.

### Determination of the activation energy of the relaxation process

**Figure 5:**
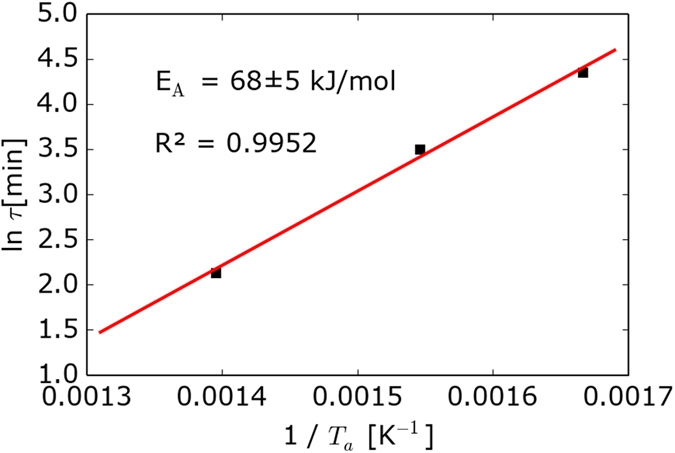
Plot of the characteristic relaxation time τ (obtained from Fig. 4) as a function of the inverse annealing temperature 1/*T*_a_. The red solid line represents a linear regression to the data points using Eq. (3), representing an activation energy of 68 kJ/mol. *R*^2^ is the coefficient of determination of the linear regression.

To obtain the activation energy of the process *E*_A_, the values of ln(τ) are plotted against the inverse annealing temperature 1/*T*_a_ in an Arrhenius-like plot of the form^26^,^28^


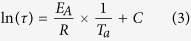


where *R* is the gas constant (*R* = 8.31 J K^−1^ mol^−1^) and *C* is an offset. Although only three data points (annealing temperatures) were available, our results strongly suggest a linear relationship between ln(*τ*) and 1/*T*_*a*_, indicative of an activation energy-mediated relaxation process. In this case, an activation energy-mediated relaxation process is the temperature-activated migration of the lightest ions within the glass network, i.e., the lithium ions. From the slope of the red line of Fig. 5, we find an activation energy of *E*_A_ = 68 ± 5 kJ/mol. which is approx. 26% smaller than the 91.2 ± 7 kJ/mol reported by Zhang *et al*.^29^ for the activation energy of lithium ion mobility in silicate melts.

The interdependency between the evolution of the Raman band N (associated with Si-O* stretching vibrations in Q^3^-structural elements) and Li migration lies in the chemical composition of the LS glass. Here, the non-bridging oxygen atom contained in each Q^3^-structure will ionically link to a lithium ion, forming a local Si-O-Li arrangement. This implicitly couples Si-O* structure and Li ion migration.

### Fs-laser induced refractive index changes

The relaxation investigations have shown that modifications of the short range order of Q^3^-structural elements (Si-O*Li in the LS glass) can be annealed at temperatures below *T*_*g*_. To investigate the influence of this annealing behaviour on the fs-laser induced refractive index changes, line-shaped optical phase objects were generated inside the multicomponent glass LNMS. The Raman spectra recorded in the centre of each phase object were treated as mentioned before and compared to the spectra of the pristine glass as a reference. Due to the lower amount of network formers of the LNMS glass in comparison with LS glass, changes of the Si-O-Si bond angle (band B) are less pronounced than in the LS glass, but changes of the Si-O* (Q^3^-stretching vibrations – band N) were found to be very similar to the changes observed in the LS glass (compare Fig. 1b and a, middle and bottom).

**Figure 6:**
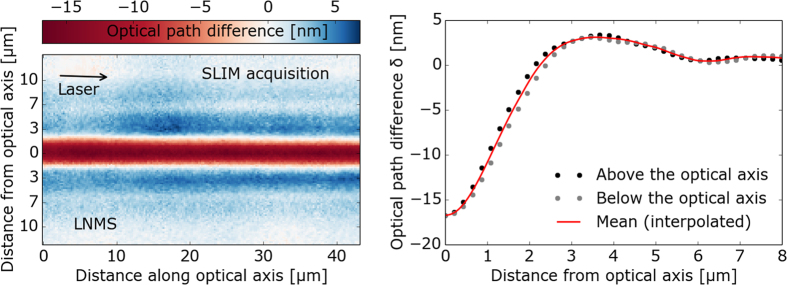
Left: Measurement of the fs-laser induced optical path difference inside the volume of a LNMS sample with the help of a spatial light interference microscope (SLIM). Right: Interpolated radial optical path difference.

To quantify the change of refractive index in LNMS glass upon fs-laser irradiation, we applied the following procedure. First, the irradiated sample was characterized with a spatial light interference microscopy SLIM apparatus (for details refer to the Method section below), providing the two-dimensional map of the radial optical path difference along the optical axis [*δ(r, z*)] as shown in Fig. 6, left. The radial optical path difference *δ(r*) through the phase object (red line in Fig. 6, right) was determined by (i) projecting *δ(r, z*) onto the *r*-axis, (ii) isolating the bottom and top parts of the line-shaped optical structure (grey and black dots in Fig. 6 right, respectively), and (iii) interpolating with 3^rd^-orders polynomials between data points. We emphasize that *δ(r*) is mostly negative in the laser modified regions and has a minimum in the centre of the modification (at *r* = 0). With increasing distance from the centre, *δ(r*) decreases and becomes positive at the edges of the phase object before approaching the value of unmodified glass [*δ(r*) = 0].

**Figure 7:**
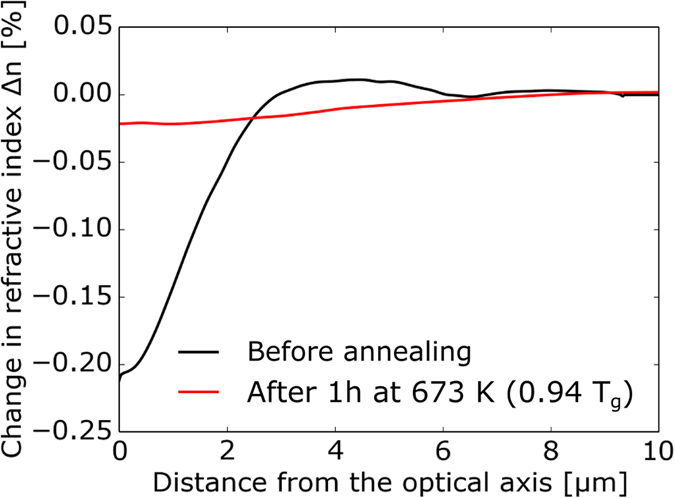
Local changes in the refractive index of the laser-induced phase object produced in LNMS glass with a pulse energy of 33 μJ and a scan velocity of 15 μm/s before (black line) and after thermal annealing at 0.94 × *T*_g_ (673 K) for 60 min (red line).

Finally, the radial dependence of the fs-laser induced refractive index Δ*n(r*) was obtained from the interpolated signal *δ(r*) by an inverse Abel transform (black line in Fig. 7). The same procedure was applied to the sample after annealing at 673 K (0.94 × *T*_g_) for 1 hour. The result (represented as a red line in Fig. 7) demonstrates that the change in refractive index almost completely vanished upon thermal annealing.

**Figure 8:**
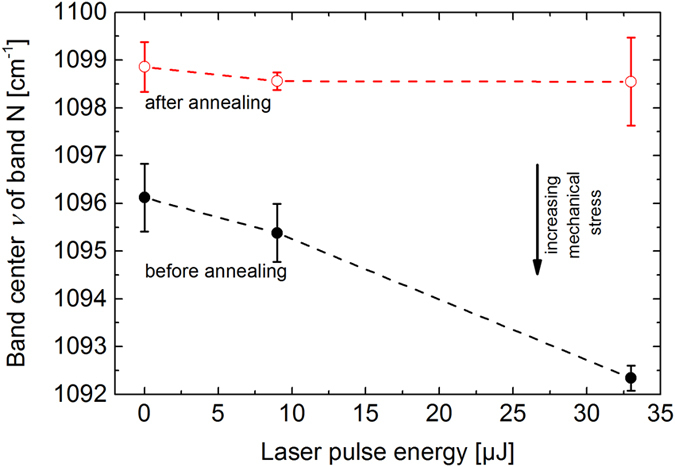
Raman spectroscopic changes of the centre position of band N (Si-O* Q^3^ stretching vibrations) before (full black circles) and after (open red circles) thermal annealing (0.94 × *T*_g_ = 673 K for 60 min) in dependence of the fs-laser pulse energy used for fabrication of the phase object in LNMS glass.

With the aim to correlate refractive index change and structural modification, we supplemented the refractive index studies with a Raman analysis of the band N centre position (see Fig. 8). As the laser pulse energy increases up to 33 μJ, the centre position of the N band shifts from 1096 cm^−1^ to 1092.5 cm^−1^ (see full black circles in Fig. 8). After the thermal annealing procedure at 0.94 × *T*_g_ for 1 hour, the band centre position also shows a relaxation up to the value of the non-irradiated glass at ~1098.5 cm^−1^ (open red circles in Fig. 8). This observation is further supported by the study of spectral changes after irradiation and successive annealing in Fig. 1b (middle and bottom), where the narrow peak, corresponding to the laser-induced modification of the band N characteristics, vanishes after thermal treatment. Note that there is a shift of ~3 cm^−1^ in the band N centre position of the Raman spectra of the non-irradiated glass recorded before and after the thermal annealing (compare the red and black circles at *F* = 0 J/cm^2^). This shift can be attributed to the presence of residual mechanical stress originating from the glass manufacturing procedure, despite the initial thermal annealing treatment at 10 K above *T*_g_ and subsequent slow cooling (see the section “Methods” below). Additional stress arises from the fs-laser modification, resulting in a further negative shift of the band N centre position for *F* > 0 J/cm^2^ (see the black data points). Both stress contributions are removed upon the second thermal annealing step. These results clearly evidence a direct relation between structural changes (causing stress) and fs-laser induced refractive index change.

## Discussion

The following scenario can be outlined for the ultrashort laser pulse induced structural modification of lithium silicate glasses: upon fs-laser pulse irradiation, the silicate glass material is transiently heated, triggering laser-induced melting allowing local re-coordination of ions and migration into the surrounding of the laser-processed spot. During the following rapid cooling stage, the material “freezes” in its non-equilibrium state, resulting in a permanently modified material in the irradiated region. In our thermal annealing experiments, this altered material was then thermally activated and relaxed toward an equilibrium state again.

The thermal annealing experiments reveal that the structural changes of the *non-bridging oxygen ions* induced by a single fs-laser pulse in the ablative regime can be successfully annealed at temperatures slightly below *T*_g_. They show, therefore, a *β*-relaxation behaviour^25^,^30^. The characteristic relaxation time *τ* depends on the annealing temperature *T*_*a*_. Measurements carried out for three distinct annealing temperatures provided *τ* values corresponding to an activation energy of *E*_A_ = 68 kJ/mol. The latter is in reasonable agreement with literature values for the migration of lithium ions in silicate melts^29^. Identifying the mobility of network modifiers as an important factor to explain the nature of the structural alterations following fs-laser irradiation opens a promising route for tailoring the fs-laser sensitivity of glasses through structural mobility.

Also, negative changes of the optical refractive index inside the glass volume after exposure to fs-laser pulses can almost completely be annealed at temperatures below *T*_g_. This result establishes that the Si-O-Si bond angle and refractive index change Δ*n* are not directly linked. Instead, we have established a direct correlation between the laser-induced Δ*n* and the configuration of the non-bridging oxygen ions. The differentiation between a glass and a liquid through structural parameters remains a standing issue. As for relaxation, it is now widely understood that above *T*_g_, i.e., in the supercooled liquid state, stress relaxation may occur through vibrational movement while in the glassy state below *T*_g_ stress relaxation requires translational movement. This could be taken as a direct reason for the shifts observed on the N- and B-bands, respectively.

## Methods

### Manufacturing of lithium silicate glasses

The binary lithium silicate glass consisting of 24 mol% Li_2_O and 76 mol% SiO_2_ (LS) and the multicomponent glass consisting of 13 mol% Li_2_O, 15 mol% Na_2_O, 9 mol% MgO and 63 mol% SiO_2_ (LNMS) were prepared by mixing reagent grade raw materials of Li_2_CO_3_ Na_2_CO_3_, MgCO_3_ and SiO_2_. The mixture was melted in a platinum crucible and kept in the liquid state for 1 hour. The glass melt was quenched between two steel plates. Subsequently, the solidified material was annealed at a temperature approximately 10 K above *T*_g_ for 60 min followed by a slow cooling at 1–2 K/min to remove residual mechanical stress inside the glass caused by the rapid quenching during the manufacturing process. This temperature was intentionally chosen very close to *T*_g_ in order to avoid crystallization. The fabricated glass plates were cut into pieces of 20 × 20 × 2 mm^3^ and polished with water free lubricant to avoid a possible modification of the glass surface. The polishing process assured an optical quality grade of the glass surface. The glass composition was determined by chemical analyses based on X-ray fluorescence and inductively coupled plasma optical emission spectrometry. Characterization of the thermophysical properties, i.e., the linear expansion coefficient (*α*) and *T*_g_ was performed by dilatometric measurements. The refractive index of the samples was calculated from the glass composition using the model of Priven^31^,^32^. The glass properties of LS and LNMS are compiled in Table 2 together with the measured mass density (*ρ*), determined experimentally by the Archimedes principle. The obtained values fit well with data available in the literature^33^.

### Fs-laser irradiation experiments

The LS glass sample surfaces were irradiated by single fs-laser pulses from a Ti:sapphire regenerative laser amplifier system (Spectra Physics GmbH, Spitfire) emitting pulses at a 800 nm centre wavelength and with ~130 fs pulse duration. The laser pulse energy was controlled by a combination of a half-wave plate and a linear polarizer. The fs-laser pulses were then focused by a spherical lens (*f* = 80 mm) yielding a Gaussian-like beam profile with a 1/e^2^-beam radius w_0_~16.5 μm at the sample surface. Laser peak fluences *F* were determined from the laser pulse energy measurements according to a method proposed by Liu^34^. The irradiation peak fluence of the laser pulses was reduced in 12 steps from ~22.5 J/cm^2^ down to 8.2 J/cm^2^. Below that fluence, no surface modification was detectable by optical microscopy. For each fluence value, the sample was irradiated by a single laser pulse on a fresh spot. At the given experimental conditions, smooth ablation craters with diameters between 4 μm and 28 μm and depths up to 350 nm were generated on the glass surfaces. All irradiations were performed in air and the spots were separated by more than 90 μm to avoid any spatial overlap.

Volume modifications were generated by focusing a fs-laser beam (200 fs pulse duration, 33 μJ pulse energy, 1 kHz repetition rate) through a 50× long working distance (LWD) objective (NA = 0.45) inside the bulk of a LNMS sample. The sample was scanned longitudinally with respect to the direction of the laser propagation with a constant velocity of 15 μm/s, resulting in line-shaped optical structures. The refractive index of those fs-laser induced structures was measured using a spatial light interference microscopy (SLIM)^35^ setup which has been used before by the authors to characterize fs-laser induced refractive index changes in fused silica^36^.

### Sample characterization

In order to perform micro-Raman analyses in the fs-laser irradiated regions, the glass sample was cleaved perpendicularly to the laser-induced optical structures at a distance of 4 mm from the sample edge. Micro-Raman spectra were acquired using a Horiba LabRam HR800 Vis-Spectrometer (Jobin-Yvon-Horiba, Longjumeau, France) in the centre of the surface ablation spots, in the centre of the volume modifications, and at the non-irradiated surface as reference. For that, the 473 nm line of a continuous wave diode laser was used as an excitation source. This radiation was focused on the sample surface by a long working distance microscope objective (Olympus, 100× LWD, NA = 0.8) illuminating a circular area of nominally ~0.7 μm in diameter. The backscattered radiation was collected with the same microscope objective along with a confocal aperture of 100 μm diameter. An optical grating of 1800 grooves/mm was selected in the spectrometer which was calibrated with the pronounced Raman peak of single-crystalline silicon positioned at 520.7 cm^−1^. The Raman signal scattered from the glass samples was recorded by a CCD camera for 50 seconds. The Raman spectra were acquired in the wavenumber range between 200 cm^−1^ and 1400 cm^−1^ with a resolution better than 1 cm^−1^. Given the transparency of the investigated glass samples in the visible spectral range, the Raman information depth was mainly limited by the Rayleigh length of the microscope objective (<3.4 μm).

All Raman spectra were baseline corrected to remove the instrumental background or fluorescence^37^. The spectra were then normalized to the highest signal value and deconvoluted into designated Gaussian-type band elements in a nonlinear least-squares calculation by the Levenberg-Marquardt fitting method^38^. Table 1 lists the characteristic Raman bands in silicate glasses used for deconvolution of the spectra. For completeness, all typical bands generally appearing in the spectra of silicate glass are listed, even if they are not selected for deconvolution within this work. For a detailed discussion of the origin for each band, please refer to the references provided in the table.

Based on the findings of our previous fs-laser experiments on glasses^22^, structural relaxation effects were analysed by annealing the modified samples at temperatures below and above *T*_g_ using a muffle furnace. After each temperature treatment, Raman spectra were acquired again in the laser-modified regions and deconvoluted as described above to reveal structural relaxation phenomena. The annealing temperatures (*T*_a_) and times (*t*_a_) are compiled in Table 3. Additionally, the change in refractive index of the sample LNMS was quantified using the SLIM-setup after annealing at 0.94 × *T*_g_ for 60 minutes.

## Additional Information

**How to cite this article:** Seuthe, T. *et al*. Structural relaxation phenomena in silicate glasses modified by irradiation with femtosecond laser pulses. *Sci. Rep.* **7**, 43815; doi: 10.1038/srep43815 (2017).

**Publisher's note:** Springer Nature remains neutral with regard to jurisdictional claims in published maps and institutional affiliations.

## Supplementary Material

Supplementary Information

## Figures and Tables

**Table 1:** Typical Raman bands used for deconvolution of silicate glass spectra along with the associated structure, origin and reference literature.

Raman Peak	Frequency Range [cm^−1^]	Structure	Origin according to literature	References
A	300–380	—	controversial	19, 39, 40, 41, 42
B	430–500	SiO_2_/Q^4^	Si-O-Si bending	19, 24, 40, 41, 42, 43, 44
C	490	D1 defect	4-membered rings of SiO_4_ tetrahedra	20, 24, 40, 43, 44
D	490–610	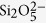 /Q^3^	—Si-O* bending	19, 24, 40, 43
E	605	D2 defect	3-membered rings of SiO_4_ tetrahedra	20, 40, 44, 45
F	590–640	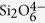 /Q^2^	–Si–O* bending	19, 40, 41
G	649–690	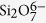 /Q^1^	-Si—O* bending	19
H/H’	770–790/810–830	SiO_2_/Q^4^	Si vibration in an oxygen cage	19, 40, 41, 42, 43, 44
I	850–890	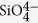 /Q^0^	Si—O* stretching	40, 41
J	870–910	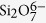 /Q^1^	-Si—O* stretching	40, 41, 46
K	940–980	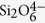 /Q^2^	–Si–O* stretching	19, 24, 40
L	1010–1060	SiO_2_/Q^4^	Si-O-Si antisymmetric stretch (TO mode)	40, 41, 43, 44
M	1000–1040	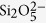 /Q^3’^	—Si-O* stretching derivative	46, 47, 48
N	1050–1100	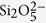 /Q^3^	—Si-O* stretching	19, 24, 40, 41
O	1150	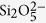 /Q^3”^	—Si-O* stretching derivative	40
P	1150–1200	SiO_2_/Q^4^	Si-O-Si antisymmetric stretch (LO mode)	19, 24, 40, 41

**Table 2:** Thermophysical properties transformation temperature (*T*_g_), mass density (*ρ*), coefficient of thermal expansion (*α*) and refractive index (*n*_D_) of the investigated glasses LS and LNMS along with the quantity of their main components as measured by chemical analysis.

Property	*T*_g_	ρ	α × 10^−6^	*n*_D_	Composition [mol%]			
Glass/Unit	K	g/cm^3^	K^−1^	—	SiO_2_	Li_2_O	Na_2_O	MgO
LS	740	2.303	9.0	1.519	76.3	23.3		
LNMS	694	2.473	13.6	1.528	62.8	12.9	15.2	8.6

**Table 3:** Set of thermal annealing experiments for investigation of the structural relaxation behaviour of the two fs-laser-irradiated silicate glasses (LS and LNMS) with annealing temperature (*T*_a_), temperature ratio (*T*_*a*_/*T*_g_) and annealing times (*t*_a_).

	*T*_a_ [K]	*T*_*a*_/*T*_g_	*t*_a_ [min]
LS A	600	0.81	10, 30, 60, 120, 240
LS B	647	0.87	10, 30, 60, 120, 240
LS C	717	0.97	10, 30, 60, 120, 240
LNMS	673	0.94	60
