# Supplementary material for: Structural relaxation phenomena in silicate glasses modified by irradiation with femtosecond laser pulses
Source: Sci Rep. 2017 Mar 7;7:43815. doi: 10.1038/srep43815 (PMC5339791; doi:10.1038/srep43815)
Supplement: Supplementary Information [file srep43815-s1.pdf]

Supplementary Material

Title Page

Title of the Manuscript

Structural relaxation phenomena in silicate glasses modified by irradiation with femtosecond laser pulses

Author List

Thomas Seuthe
Alexandre Mermillod-Blondin
Moritz Grehn
Jörn Bonse
Lothar Wondraczek
Markus Eberstein

Correspondence and requests for materials should be addressed to Markus Eberstein (markus.eberstein@ikts.fraunhofer.de)

supplementary_material

October 17, 2016

```
In [1]: %matplotlib inline
        import numpy as np
        import matplotlib.pyplot as plt
        import matplotlib as mpl
        from scipy import optimize
        from scipy.optimize import curve_fit
```

```
In [2]: mpl.rcParams['font.size'] = 16
```

1 1. Reproduce Fig. 3

1.1 a. Define time vectors and corresponding slopes

```
In [3]: #timevector for the experiments at Ta = 0.7 x Tg:
        ta_s_07Tg = np.array([1.,600,1800,3600,7200,14400]) # in seconds
        ta_min_07Tg = ta_s_07Tg / 60 # in minutes
        #Estimated slopes at 0.7 x Tg for different times:
        s_ta_07Tg = np.array([-0.057, -0.048, -0.033, -0.029, -0.009, -0.005])
        #Fit uncertainties on the slopes
        yerr_s_ta_07Tg = [0.007, 0.007, 0.006, 0.003, 0.002, 0.007]

        #timevector for the experiments at Ta = 0.8 x Tg:
        ta_s_08Tg = np.array([1., 1800,3600,7200]) # in seconds
        ta_min_08Tg = ta_s_08Tg / 60 # in minutes
        #Estimated slopes at 0.8 x Tg for different times:
        s_ta_08Tg = np.array([-0.10, -0.066, -0.002, -0.002])
        #Fit uncertainties on the slopes
        yerr_s_ta_08Tg = [0.006, 0.012, 0.013, 0.004]

        #timevector for the experiments at Ta = 0.95 x Tg:
        ta_s_095Tg = np.array([1.,600,1800,3600,7200]) # in seconds
        ta_min_095Tg = ta_s_095Tg / 60 # in minutes
        s_ta_095Tg = np.array([-0.081, -0.032, -0.002, -0.002, -0.003])#cf Table1 in file
        #Fit uncertainties on the slopes:
        yerr_s_ta_095Tg = [0.007, 0.006, 0.005, 0.028, 0.018]
```

1.2 b. Plot of S(t_a) vs annealing time

```
In [4]: ###############################################################################################
        #plot
        ###############################################################################################
        fig = plt.figure(figsize=(6, 4))
        ax = fig.add_subplot(111)
```

```
line1, capline1, barline1 = ax.errorbar(ta_min_07Tg, s_ta_07Tg, yerr=yerr_s_ta_07Tg,
                                        fmt='s', color='black')
ax.set_xlim((-10, np.max(ta_min_07Tg)+10))
line2 = ax.plot(ax.get_xlim(), [0,0], ls='--', lw=2, color='gray')

ax.set_xlim((-10, np.max(ta_min_07Tg)+10))
ax.set_xlabel('Annealing time $t_{a}$ [min.]')
ax.set_ylabel('$S(t_{a})$ of band N [cm/J]')
ax.legend(['Ta = 0.7 x Tg'], loc='lower right', frameon=False)
```

Out[4]: <matplotlib.legend.Legend at 0x7f7fa130e5d0>

```
/usr/lib/pymodules/python2.7/matplotlib/collections.py:548: FutureWarning: elementwise comparison failed
  if self._edgecolors == 'face':
```

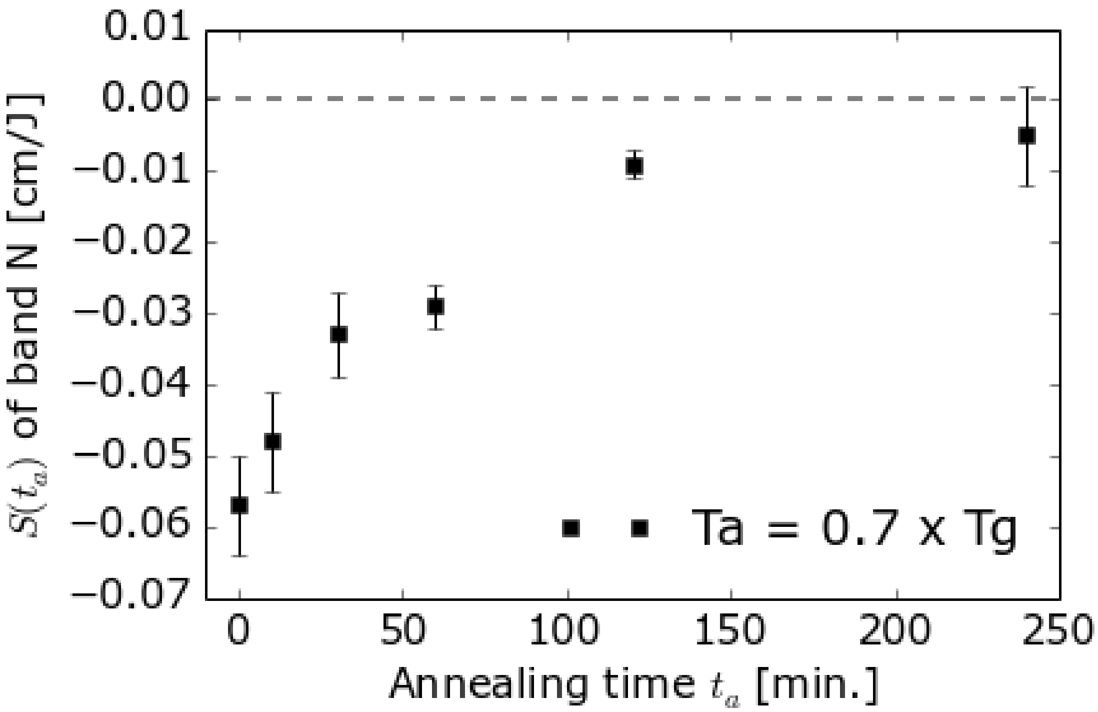

2 2. Generate Fig. 4

2.0.1 a. Compute $\xi(t_a)$'s

In [5]:
```
#Compute ξ(ta) from S(ta) using Eq. 1 with S(∞) = 0 (see paragraph Structural relaxation below
xhi_ta_07Tg = (s_ta_07Tg - 0) / (s_ta_07Tg[0] - 0)
xhi_ta_08Tg = (s_ta_08Tg - 0) / (s_ta_08Tg[0] - 0)
xhi_ta_095Tg = (s_ta_095Tg - 0) / (s_ta_095Tg[0] - 0)
```

2.0.2 b. Compute uncertainties on $\xi(t_a)$'s

The variables s(ta) and s(0) are physically uncorrelated. Therefore, formula which applies is:

$\sigma_{\xi(ta)} = \langle\xi(t_a)\rangle\sqrt{(\frac{\sigma_{ta}}{S_{ta}})^2 + (\frac{\sigma_0}{S_0})^2}$,

with σ corresponding to the square root of the variance and $\langle\rangle$ corresponding to the mean value of the variable.

In [6]:
```
####################################################################################
#estimate uncertainties using the formula given above
####################################################################################

xhi_ta_07Tg_err = xhi_ta_07Tg * np.sqrt((yerr_s_ta_07Tg / s_ta_07Tg) ** 2 + (
        yerr_s_ta_07Tg[0] / s_ta_07Tg[0]) ** 2)
xhi_ta_08Tg_err = xhi_ta_08Tg * np.sqrt((yerr_s_ta_08Tg / s_ta_08Tg) ** 2 + (
        yerr_s_ta_08Tg[0] / s_ta_08Tg[0]) ** 2)
xhi_ta_095Tg_err = xhi_ta_095Tg * np.sqrt((
        yerr_s_ta_095Tg / s_ta_095Tg) ** 2 + (yerr_s_ta_095Tg[0] / s_ta_095Tg[0]) ** 2)
```

2.0.3 c. Compute fit parameters

Fit $\xi(t_a)$ with Eq. 2: exp $[\ t_a\ /\ \tau]^{\ 0.6}$ (only 1 fit parameter: τ).

In [7]:
```
#definition of the fit function, where tau is the only fit parameter
fitfunc_tau = lambda xdata, tau: np.exp(-(xdata / tau) ** 0.6)

######################################################################################
# Compute tau values. The fit method curve_fit is a wrapper around
#MINPACK's lmdif and lmder algorithms.
######################################################################################
#07Tg:
tau_guess_07Tg = [10000.]
tau_07Tg, pcov_07Tg = curve_fit(f=fitfunc_tau, xdata=ta_s_07Tg, ydata=xhi_ta_07Tg,
                                p0=tau_guess_07Tg)

#08Tg:
tau_guess_08Tg = [100.]
tau_08Tg, pcov_08Tg = curve_fit(f=fitfunc_tau, xdata=ta_s_08Tg, ydata=xhi_ta_08Tg,
                                p0=tau_guess_08Tg)


#095Tg:
tau_guess_095Tg = [500.]
tau_095Tg, pcov_095Tg = curve_fit(f=fitfunc_tau, xdata=ta_s_095Tg, ydata=xhi_ta_095Tg,
                                  p0=tau_guess_095Tg)
```

(i) Plot results and fit. One graph per annealing temperature.

In [8]:
```
####################################################################################
#plot results corresponding to each annealing temperature on a different graph
####################################################################################

fig = plt.figure(figsize=(16, 4))

#define common template for all graphs
def plot_xhi_and_fit(ax, time_vector, xhi, yerr, fitfunc, tau, pcov):
    tmin = 0.1
    tmax = 100000
    timefit = np.logspace(np.log10(tmin), np.log10(tmax))
```

```
    linefit = ax.plot(timefit, fitfunc(timefit, tau), lw='2', ls='--', color='black')
    line, capline, barline = ax.errorbar(time_vector, xhi, yerr, fmt='s', color='black')
    ax.set_xscale('log')
    ax.set_xlabel('Annealing time $t_{a}$ [s]')
    ax.set_ylabel(u'ξ$(t_{a})$ of band N [no units]')
    ax.set_xlim(0.7, 100000)
    ax.set_ylim(-0.4, 1.2)
    ax.legend([r'$\tau$ = %i$\pm$%i s' % (tau, np.sqrt(pcov[0]))], frameon=False,
              loc='lower left', fontsize=16)

ax1 = fig.add_subplot(131)
plot_xhi_and_fit(ax1, ta_s_07Tg, xhi_ta_07Tg, xhi_ta_07Tg_err, fitfunc_tau, tau_07Tg,
                 pcov_07Tg)
ax1.set_title('$T_a$ = 0.7 x $T_g$')

ax2 = fig.add_subplot(132)
plot_xhi_and_fit(ax2, ta_s_08Tg, xhi_ta_08Tg, xhi_ta_08Tg_err, fitfunc_tau, tau_08Tg,
                 pcov_08Tg)
ax2.set_title('$T_a$ = 0.8 x $T_g$')

ax3 = fig.add_subplot(133)
plot_xhi_and_fit(ax3, ta_s_095Tg, xhi_ta_095Tg, xhi_ta_095Tg_err, fitfunc_tau, tau_095Tg,
                 pcov_095Tg)
ax3.set_title('$T_a$ = 0.95 x $T_g$')

plt.tight_layout()
```

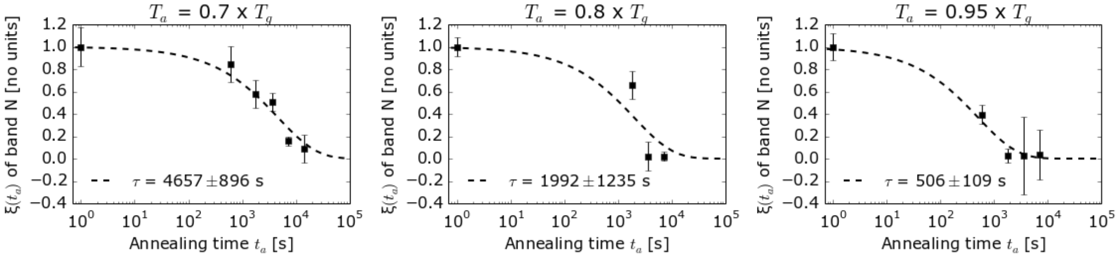

In [9]:
```
##############################################################################
# generate fig. 4 by plotting all results on same graph
##############################################################################

#define routine to customize the aspect of the data points and error bars
def set_aspect(line, caplines, barlines, color):
    "sets color and transparency for error bars"
    alpha= 0.7

    line.set_color(color)
    line.set_alpha(alpha)
    line.set_markersize(8)

    for cap in caplines:
        cap.set_color(color)
```

```
        cap.set_markeredgewidth(1)
        cap.set_alpha(alpha)

    for bar in barlines:
        bar.set_color(color)
        bar.set_linewidth(1.)
        bar.set_alpha(alpha)

#Prepare figure:
tmin = 1.
tmax = 100000
timefit = np.logspace(np.log10(tmin), np.log10(tmax))

fig = plt.figure(figsize=(6, 4))
ax = fig.add_subplot(111)

ax.set_xscale('log')
ax.set_xlabel('Annealing time $t_{a}$ [s]')
ax.set_ylabel(u'ξ$(t_{a})$')
ax.set_xlim(0.8, 100000)
ax.set_ylim(-0.4, 1.3)

#plot data with errorbars:
#at 07xTg:
line1, capline1, barline1 = ax.errorbar(ta_s_07Tg, xhi_ta_07Tg, yerr=xhi_ta_07Tg_err,
                                        fmt='s', color='black')
set_aspect(line1, capline1, barline1, 'black')
#at 08xTg:
line2, capline2, barline2 = ax.errorbar(ta_s_08Tg, xhi_ta_08Tg, yerr=xhi_ta_08Tg_err,
                                        fmt='o', color='red')
set_aspect(line2, capline2, barline2, 'red')
#at 095xTg:
line3, capline3, barline3 = ax.errorbar(ta_s_095Tg, xhi_ta_095Tg, yerr=xhi_ta_095Tg_err,
                                        fmt='^', color='blue')
set_aspect(line3, capline3, barline3, 'blue')

#plot fits:
#at 07xTg:
line1_fit, = ax.semilogx(timefit, fitfunc_tau(timefit, tau_07Tg), lw='2', ls='--',
                         color='black')
#at 08xTg:
line2_fit, = ax.semilogx(timefit, fitfunc_tau(timefit, tau_08Tg), lw='2', ls='--',
                         color='red')
#at 095xTg:
line3_fit, = ax.semilogx(timefit, fitfunc_tau(timefit, tau_095Tg), lw='2', ls='--',
                         color='blue')

#make legend; a multiple legend provides more information whereas preserving readability:
leg1 = ax.legend([line1, line2, line3], [u'0.7 $T_g$', u'0.8 $T_g$', u'0.95 $T_g$'],
                 loc='upper right', frameon=False, fontsize=14, numpoints=1)
ax.add_artist(leg1) #because only one legend is allowed,
                    #we need to incorporate the previous artist to the axis.
```

```
leg2 = ax.legend([line1_fit, line2_fit, line3_fit], [r'$\tau$ = %i$\pm$%i s' %(
            tau_07Tg, np.sqrt(pcov_07Tg[0])),
                 r'$\tau$ = %i$\pm$%i s' %(tau_08Tg, np.sqrt(pcov_08Tg[0])),
                 r'$\tau$ = %i$\pm$%i s' %(tau_095Tg, np.sqrt(pcov_095Tg[0]))],
                 frameon=False, loc='lower left', fontsize=14)
```

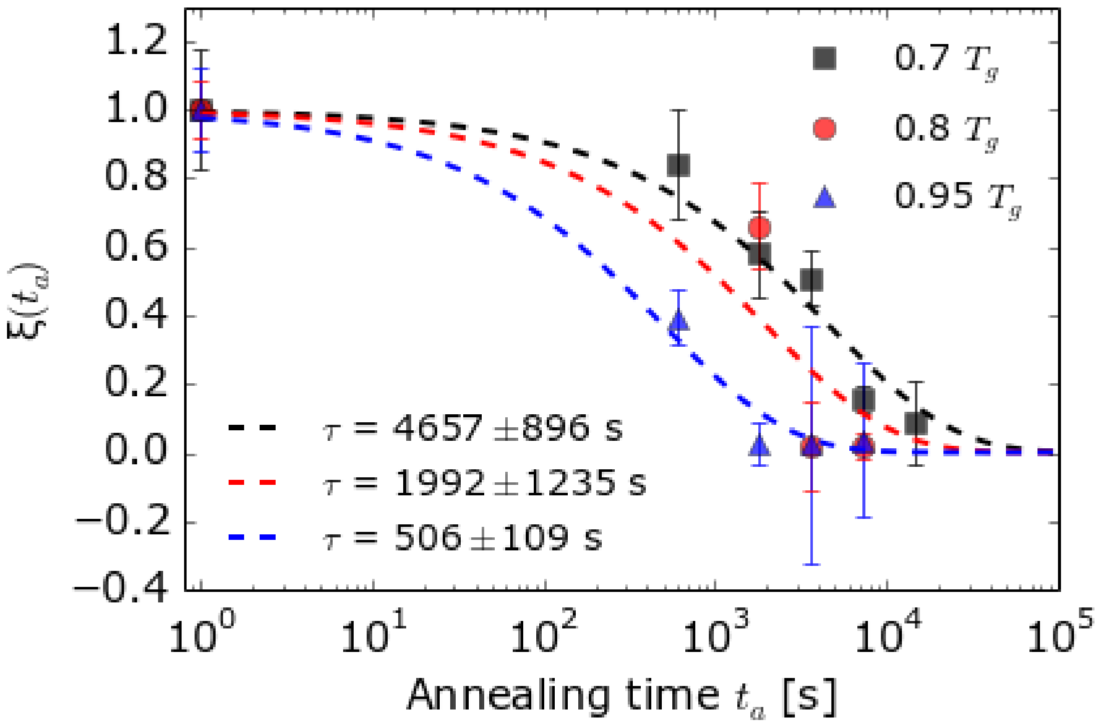

3 3. Figure 5

3.1 a. Retrieve activation energy

(i) Compute activation energy with the help of Eq. 3

In [10]:
```
##########################################################################################
#fit
##########################################################################################

#convert annealing temperatures from Celsius to Kelvin
Tg = 467 #in °C
Tannealing = np.array([1/(0.95*Tg + 273.15) , 1/(0.8*Tg + 273.15), 1/(0.7*Tg + 273.15)])
#compute ln(tau)
taus = np.array([np.log(tau_095Tg[0]/60), np.log(tau_08Tg[0]/60), np.log(tau_07Tg[0]/60)])
#lauch fit routine
fitfunc_activation_energy = lambda T_ann, p0, p1 : p0 * T_ann + p1
#fit
p, cov = curve_fit(f=fitfunc_activation_energy,
                   xdata=Tannealing, ydata=taus) #p[0] contains the best fit for the slope
```

```
#compute activation energy
R = 8.314  #Gas constant
act_energy = p[0] * R
act_energy_uncertainty = np.sqrt(cov[0,0]) * R
```

(ii) Check linear dependence between ln(τ) and 1/Ta by estimating the coefficient of determination

```
In [11]: ###########################################################################################
         # Evaluate R2
         #    see https://en.wikipedia.org/wiki/Coefficient_of_determination#Definitions
         #    see http://stackoverflow.com/questions/20115272/
         #        calculate-coefficient-of-determination-r2-and-root-mean-square-error-rmse-fo
         ###########################################################################################

         def compute_R2(xdata, ydata, func, fit_result):
             """see https://en.wikipedia.org/wiki/Coefficient_of_determination
                see http://stackoverflow.com/questions/20115272/
                calculate-coefficient-of-determination-r2-and-root-mean-square-error-rmse-fo
             """
             ymean = np.mean(ydata)
             ss_tot = np.dot((ydata - ymean), (ydata - ymean))
             ss_res = np.dot((ydata - func(xdata, fit_result[0], fit_result[1])),
                             (ydata - func(xdata, fit_result[0], fit_result[1])))
             r2 = (1 - ss_res / ss_tot)
             return r2

         r_square = compute_R2(Tannealing, taus, fitfunc_activation_energy, p)
```

(iii) Plot figure 5

```
In [12]: ###########################################################################################
         #plot
         ###########################################################################################
         fig = plt.figure()
         ax = fig.add_subplot(111)

         Tann_fit = np.linspace(0.00131, 0.00169)
         ln_tau_fit = fitfunc_activation_energy(Tann_fit, p[0], p[1])

         #plot points
         line1, = ax.plot(Tannealing, taus, marker='s', ls='none', color='black')
         #plot best fit
         linefit, = ax.plot(Tann_fit, ln_tau_fit, lw=2, color='red')

         #customize plot
         ticks = ax.set_xticks([0.0013, 0.0014, 0.0015, 0.0016, 0.0017])
         ax.set_xlim((0.0013, 0.0017))
         xtitle = ax.set_xlabel(u'1 / $T_a$ [K$^{-1}$]')
         ytitle = ax.set_ylabel(r'ln $ \tau $[min]')

         textbox = ax.text(0.00135, 3.5,  u'E$\mathrm{_A}$ = %i±%i kJ/mol\n\nR² = %3.4f' % (
                 np.round(act_energy / 1000), np.round(act_energy_uncertainty / 1000), r_square))
```

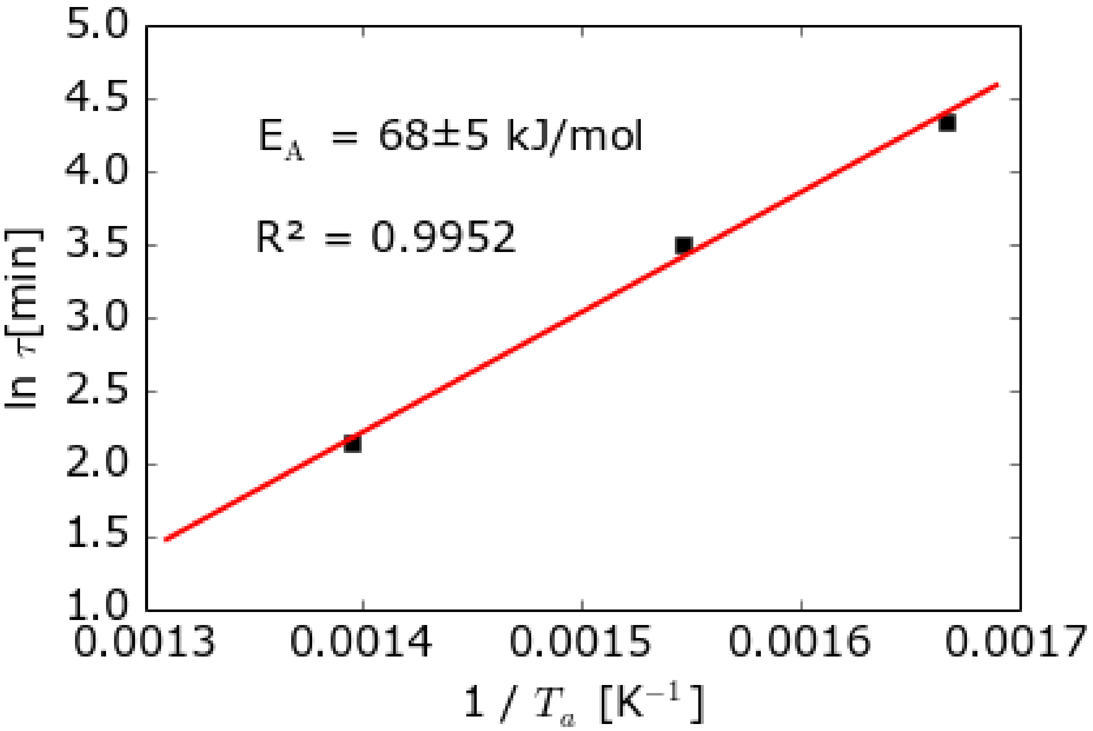

E$_A$ = 68±5 kJ/mol
R² = 0.9952
ln τ[min]
1 / T_a [K^{-1}]
5.0
4.5
4.0
3.5
3.0
2.5
2.0
1.5
1.0
0.0013
0.0014
0.0015
0.0016
0.0017

In [ ]:
